# A randomized controlled study on intermittent theta pulse stimulation for improving cognitive impairment after stroke

**DOI:** 10.3389/fneur.2025.1608639

**Published:** 2025-08-19

**Authors:** Fei Li, Fengxia Hu, Yi Liang, Fen Liang, Huiqun Tan, Sisi Xing

**Affiliations:** ^1^Department of Neurology, Huangshi Central Hospital, Affiliated Hospital of Hubei Polytechnic University, Huangshi, Hubei, China; ^2^Department of Gastroenterology, Huangshi Central Hospital, Affiliated Hospital of Hubei Polytechnic University, Huangshi, Hubei, China; ^3^Department of General Practice, Huangshi Central Hospital, Affiliated Hospital of Hubei Polytechnic University, Huangshi, Hubei, China

**Keywords:** intermittent theta-burst stimulation (iTBS), stroke, cognitive dysfunction, neurorehabilitation, quality of life

## Abstract

**Objective:**

This study evaluates the efficacy and underlying mechanisms of intermittent theta-burst stimulation (iTBS) in improving cognitive function and quality of life in post-stroke patients.

**Methods:**

A total of 80 subacute stroke patients with cognitive deficits were randomly assigned to a control group (*n* = 40) receiving conventional treatment plus sham stimulation and an experimental group (*n* = 40) receiving conventional treatment plus iTBS over the left dorsolateral prefrontal cortex for 4 weeks.

**Results:**

Baseline characteristics were comparable between groups. After 3 months, the experimental group demonstrated significantly greater improvements than the control group in scores for the Mini-Mental State Examination (MMSE; adjusted mean: 25.35 vs. 20.44, *P* < 0.001), Montreal Cognitive Assessment (MoCA; 26.49 vs. 24.57, P = 0.002), and Stroke-Specific Quality of Life (SS-QOL; 158.45 vs. 137.31, *P* < 0.001), and showed greater reduction in completion time for the Trail Making Test (TMT). Biochemically, the iTBS group exhibited significantly increased serum Brain-Derived Neurotrophic Factor (BDNF) and reduced levels of Tumor Necrosis Factor-alpha (TNF-α) and Interleukin-6 (IL-6) compared to the control group (all P < 0.001). Changes in BDNF levels correlated positively with improvements in MMSE scores (*r* = 0.58, *P* < 0.001).

**Conclusion:**

iTBS is a safe and effective intervention that enhances cognitive recovery and quality of life in post-stroke patients. These benefits are associated with modulation of neuroplasticity and inflammatory markers, suggesting that iTBS may promote recovery by upregulating BDNF and attenuating neuroinflammation. Further research is needed to confirm these mechanisms.

## Introduction

Traditional post-stroke rehabilitation methods, including drug therapy, physical therapy, and cognitive training (1–3), often yield limited benefits due to individual variability in treatment response (4, 5). Non-invasive brain stimulation techniques, such as intermittent theta-burst stimulation (iTBS), have emerged as promising alternatives for enhancing neuroplasticity and functional recovery (6–8). While systematic reviews highlight iTBS efficacy in improving motor deficits post-stroke (9, 10), evidence supporting its role in cognitive rehabilitation remains fragmented.

iTBS, a patterned form of transcranial magnetic stimulation, modulates neuronal excitability through high-frequency bursts, potentially accelerating neuroplasticity (11). Neuroplasticity—the brain's ability to reorganize networks after injury—is critical for restoring cognitive functions such as attention, memory, and executive control (12, 13). Although preliminary studies suggest iTBS may enhance cognitive outcomes in stroke patients, existing trials are limited by small sample sizes, short follow-up periods (typically ≤ 3 months), and inconsistent outcome measures (14, 15). For instance, a recent systematic review (1) identified only six randomized controlled trials (total *n* = 312) investigating iTBS for post-stroke cognitive impairment, with heterogenous protocols and inconclusive long-term benefits.

This study addresses these gaps by evaluating iTBS effects on cognitive function and quality of life in a rigorously controlled trial with a 3-month follow-up. We selected a comprehensive battery of assessments: the Mini-Mental State Examination (MMSE) and Montreal Cognitive Assessment (MoCA) for global cognition, the Trail Making Test (TMT) for executive function, and the Stroke-Specific Quality of Life (SS-QOL) scale for functional wellbeing. To explore underlying mechanisms, we measured serum levels of Brain-Derived Neurotrophic Factor (BDNF), a key marker of neuroplasticity, and pro-inflammatory cytokines Tumor Necrosis Factor-alpha (TNF-α) and Interleukin-6 (IL-6), which are implicated in post-stroke neural injury and repair. By clarifying its therapeutic potential and underlying mechanisms, this work aims to advance personalized neurorehabilitation strategies for stroke survivors.

## Materials and methods

### Ethical approval and trial registration

This study was approved by the Ethics Committee of Huangshi Central Hospital. All procedures were performed in accordance with the Declaration of Helsinki, and written informed consent was obtained from all participants.

### Participants

**Figure 1:**
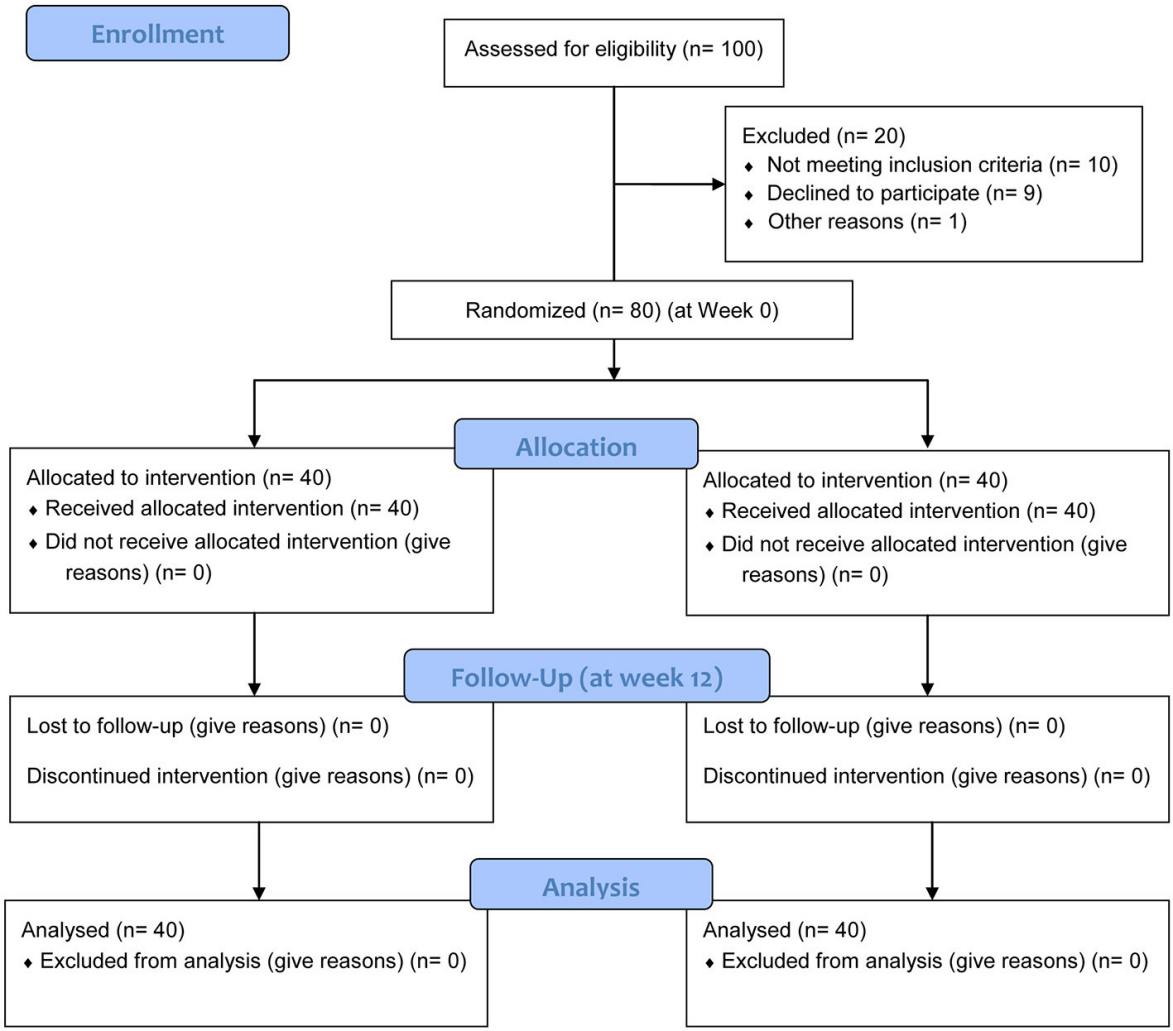
CONSORT 2010 flow diagram showing the process of patient enrollment, allocation, follow-up, and analysis.

Between March 2022 and May 2024, 100 patients were assessed for eligibility, and 80 post-stroke patients with cognitive impairment were recruited from the Department of Neurology and Rehabilitation Medicine. Inclusion criteria were: (1) Age 18–80 years; (2) Confirmed first-ever ischemic or hemorrhagic stroke (diagnosed via CT/MRI); (3) Stroke onset between 1 and 6 months prior to enrollment (subacute phase); (4) Mild-to-moderate cognitive impairment, defined as a score of 18–26 on the Montreal Cognitive Assessment (MoCA) (16); (5) Ability to understand and comply with treatment and assessment procedures. Exclusion criteria included: severe aphasia or dementia, other pre-existing neurodegenerative diseases (e.g., Alzheimer's disease, Parkinson's disease), major psychiatric disorders, a history of seizures, metallic implants in the head, pacemaker, pregnancy, or refusal to provide informed consent. A Consolidated Standards of Reporting Trials (CONSORT) flow chart showing the enrollment process is shown in Figure 1.

### Sample size calculation

The sample size was determined a priori based on a previous study by Li et al. (6), which reported a significant improvement in MMSE scores following iTBS. Assuming a mean difference of 2.5 points in MMSE change scores with a standard deviation of 3.0, a power of 80% (β = 0.20), and a two-sided alpha level of 0.05, a sample size of 30 participants per group was required. To account for a potential dropout rate of up to 20%, we aimed to recruit 40 participants per group, for a total of 80 participants.

### Randomization and blinding

Participants were randomly assigned in a 1:1 ratio to either the experimental (iTBS) or control (sham iTBS) group. The randomization sequence was generated by a computer and concealed using sequentially numbered, opaque, sealed envelopes prepared by a research assistant not involved in patient recruitment or assessment.

### Blinding

This study was conducted as a double-blind trial. Participants were blinded to their group assignment. The outcome assessors who administered the neuropsychological tests and the research staff who collected biochemical data were also blinded to group allocation. Furthermore, the statistician who performed the data analysis was blinded to the group assignments until after the primary analysis was complete.

### Interventions

Both groups received a standardized conventional therapy protocol. This included optimal medical management (antiplatelet agents, antihypertensives, and statins) as per clinical guidelines. In addition, all participants underwent a cognitive rehabilitation program for 30 min per day, 5 days a week, for 4 weeks. This program was delivered by a trained therapist and was standardized across both groups, involving structured, computer-assisted tasks targeting memory (N-back tasks), attention (sustained attention response tasks), and executive functions (Wisconsin Card Sorting Test simulation). The difficulty of the tasks was progressively increased based on individual performance to ensure continued challenge.

### Experimental group (iTBS)

**Device:** A YRD CCY-1 transcranial magnetic stimulator (Wuhan IRED Medical Devices Co., Ltd., China) with a figure-of-eight coil was used.

**Target:** The left dorsolateral prefrontal cortex (DLPFC), localized using the international 10–20 EEG system at the F3 position (17).

**Parameters:** The iTBS protocol consisted of 3-pulse bursts at 50 Hz, repeated at a 5 Hz (theta) rhythm. Stimulation was delivered in a 2-s train followed by an 8-s inter-train interval, for a total of 600 pulses per session. The stimulation intensity was set at 80% of the individual's resting motor threshold (RMT). RMT was defined as the minimum stimulator output required to elicit a motor evoked potential (MEP) of at least 50 μV peak-to-peak amplitude in at least 5 out of 10 consecutive trials from the contralateral first dorsal interosseous muscle.

**Figure 2:**
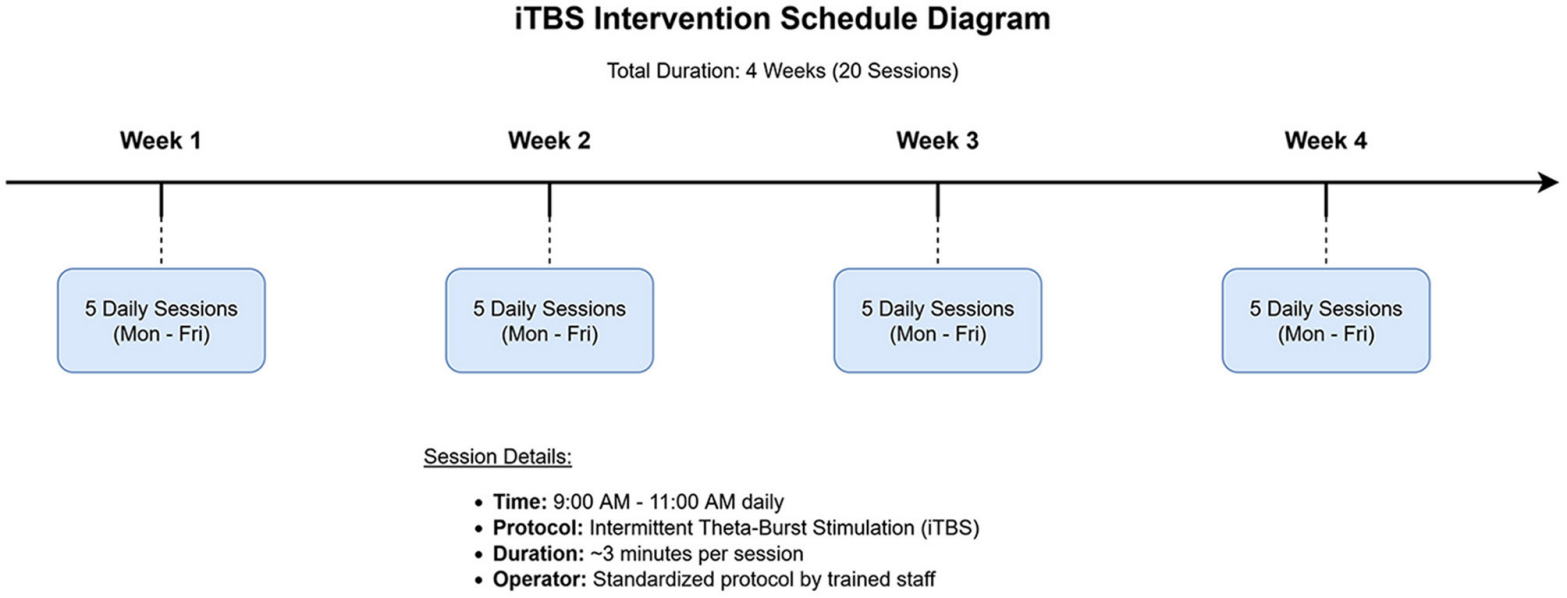
Schematic diagram of the intermittent theta-burst stimulation (iTBS) intervention schedule. The protocol spanned 4 weeks, with 5 daily sessions administered from Monday to Friday each week, for a total of 20 sessions.

**Schedule:** The iTBS protocol consisted of 20 sessions, administered once daily for 5 consecutive days per week, over a period of 4 weeks. Each iTBS session, lasting approximately 3 min and 10 s, was conducted between 9:00 AM and 11:00 AM to minimize potential circadian variations in cortical excitability. All stimulation sessions were performed by one of two trained operators (F.L., Y.L.) following a strict, standardized protocol to ensure consistency (see Figure 2 for a schematic of the intervention schedule).

### Control group (Sham)

Participants in the control group underwent an identical setup and procedure as the iTBS group. However, a sham coil was used, which was identical in appearance and produced the same auditory click as the active coil, but the coil was angled at 90° with the edge touching the scalp. This orientation ensures that the magnetic field does not effectively penetrate the scalp, thus avoiding cortical stimulation while providing similar tactile and auditory sensations to maintain blinding.

### Outcome measures

All outcomes were assessed at baseline (before intervention) and at 3 months post-intervention by a blinded assessor.

**Cognitive function:** Assessed using: (1) The Mini-Mental State Examination (MMSE) (18), a 30-point test of global cognitive function. (2) The Montreal Cognitive Assessment (MoCA) (18), a 30-point screening tool sensitive to mild cognitive impairment. (3) The Trail Making Test (TMT) (19), consisting of Part A (TMT-A), which measures processing speed, and Part B (TMT-B), which assesses cognitive flexibility and executive function. The time to complete each part was recorded in seconds.

**Quality of life:** Measured using the Stroke-Specific Quality of Life (SS-QOL) scale (20), a 49-item self-report questionnaire assessing health-related quality of life across 12 domains.

**Biochemical markers:** For biochemical analysis, venous blood samples (5 mL) were collected from each participant in the morning after an overnight fast at baseline and at the 3-month follow-up. Samples were collected in serum-separating tubes, allowed to clot for 30 min at room temperature, and then centrifuged at 3,000 rpm for 15 min at 4°C. The resulting serum was aliquoted and stored at −80°C until analysis. Serum levels of Brain-Derived Neurotrophic Factor (BDNF), Tumor Necrosis Factor-alpha (TNF-α), and Interleukin-6 (IL-6) were quantified using commercial enzyme-linked immunosorbent assay (ELISA) kits (R&D Systems, Minneapolis, MN, USA) according to the manufacturer's instructions. All samples were analyzed in duplicate by a technician blinded to group allocation, and the average value was used for analysis. The intra- and inter-assay coefficients of variation were all below 10%.

### Statistical analysis

Data analysis was performed using SPSS version 26.0 (IBM Corp., Armonk, NY, USA). Normality of data distribution was assessed using the Shapiro-Wilk test. Baseline demographic and clinical characteristics were compared between groups using independent samples *t*-tests for normally distributed continuous data, Mann-Whitney U tests for non-normally distributed continuous data, and chi-squared (χ^2^) or Fisher's exact tests for categorical data. The primary analysis of post-intervention outcomes was conducted using analysis of covariance (ANCOVA), with the 3-month outcome score as the dependent variable, group allocation (iTBS vs. sham) as the fixed factor, and baseline scores, age, and time since stroke included as covariates. Adjusted means and their standard errors (SE) are reported. Within-group changes from baseline to 3 months were assessed using paired *t*-tests or Wilcoxon signed-rank tests where appropriate. Effect sizes for ANCOVA are reported as partial eta squared (ηp^2^). In the experimental group, Pearson correlation analysis was performed to explore the relationship between the change in biochemical markers and the change in cognitive scores from baseline to 3 months. A *P*-value < 0.05 was considered statistically significant.

## Results

### Patient characteristics

Of the 100 patients screened, 80 were enrolled and randomized (40 to the iTBS group, 40 to the control group). All participants completed the study, with no dropouts (Figure 1). The two groups were well-matched at baseline with no significant differences in gender, age, Body Mass Index (BMI), stroke type, time since stroke, or prevalence of comorbidities such as hypertension, diabetes, smoking, and alcohol use (all *P* > 0.05; Table 1).

**Table 1:** Baseline demographic and clinical characteristics of participants.

**Characteristic**	**Control group (*n* = 40)**	**Experimental group (*n* = 40)**	***t*/χ^2^ value**	***P*-value**
Gender (male: female)	28:12	25:15	0.455	0.500
Age (years), mean ± SD	59.8 ± 7.2	60.5 ± 6.9	−0.431	0.668
BMI (kg/m^2^), mean ± SD	22.33 ± 1.48	23.62 ± 1.77	−3.641	0.241
Time since Stroke (months), mean ± SD	3.5 ± 1.2	3.7 ± 1.3	−0.712	0.479
**Stroke type**			0.170	0.680
- Ischemic, n (%)	27 (67.5%)	29 (72.5%)		
- Hemorrhagic, n (%)	13 (32.5%)	11 (27.5%)		
Hypertension, n (%)	8 (20.0%)	10 (25.0%)	0.357	0.550
Diabetes, n (%)	4 (10.0%)	5 (12.5%)	0.119	0.730
Smoking, n (%)	11 (27.5%)	9 (22.5%)	0.278	0.598
Alcohol use, n (%)	14 (35.0%)	13 (32.5%)	0.051	0.821

BMI, Body Mass Index. Data are presented as mean ± standard deviation (SD) or n (%). *P*-values were derived from independent samples *t*-tests for continuous variables and χ^2^ tests for categorical variables.

### Safety and tolerability

The iTBS intervention was well-tolerated by all participants in the experimental group. No serious adverse events, such as seizures or syncope, were reported. Three participants in the iTBS group reported a mild, transient headache immediately following stimulation, which resolved spontaneously within an hour and did not require medication. Two participants in the sham group reported similar mild headaches. Adherence to the treatment protocol was excellent, with all 80 participants completing all 20 intervention sessions and the 3-month follow-up assessment.

### Cognitive function, quality of life, and biochemical outcomes

At baseline, there were no significant between-group differences in any cognitive, quality of life, or biochemical measures (all *P* > 0.05). After the 3-month intervention, the ANCOVA revealed significant group effects for all outcomes. The iTBS group showed significantly greater improvements in MMSE, MoCA, and SS-QOL scores, and significantly greater reductions in TMT-A and TMT-B completion times compared to the control group (all *P* < 0.01). Furthermore, the iTBS group had significantly higher post-intervention BDNF levels and significantly lower TNF-α and IL-6 levels (all *P* < 0.001). Detailed results, including adjusted means and effect sizes, are presented in Table 2.

**Table 2:** Comparison of clinical and biochemical outcomes at baseline and 3 months.

**Measure**	**Baseline (Mean** ±**SD)**		**3 Months (Adjusted Mean** ±**SE)**		**Between-group difference (95% CI)**	**Partial η^2^**	***p*-value**
	**Control (*****n*** = **40)**	**Experimental (*****n*** = **40)**	**Control (*****n*** = **40)**	**Experimental (*****n*** = **40)**			
**Cognitive function**							
MMSE	19.35 ± 1.86	19.19 ± 2.05	20.44 ± 0.31	25.35 ± 0.31	+4.91 (3.92 to 5.90)	0.65	<0.001
MoCA	20.33 ± 1.44	20.16 ± 1.37	24.57 ± 0.28	26.49 ± 0.28	+1.92 (1.09 to 2.75)	0.28	<0.001
TMT-A Time (s)	75.45 ± 8.27	77.62 ± 9.45	72.13 ± 1.35	60.26 ± 1.35	−11.87 (−15.82 to −7.92)	0.42	<0.001
TMT-B Time (s)	136.45 ± 11.27	140.62 ± 10.45	128.33 ± 1.41	117.26 ± 1.41	−11.07 (−15.23 to −6.91)	0.35	<0.001
**Quality of life**							
SS-QOL	125.27 ± 15.41	123.69 ± 13.08	137.31 ± 1.70	158.45 ± 1.70	+21.14 (17.79 to 24.49)	0.68	<0.001
**Biochemical markers**							
BDNF (ng/mL)	24.11 ± 1.82	24.44 ± 1.79	24.25 ± 0.36	31.56 ± 0.36	+7.31 (6.27 to 8.35)	0.75	<0.001
TNF-α (pg/mL)	20.91 ± 2.39	20.62 ± 2.31	20.73 ± 0.31	13.27 ± 0.31	−7.46 (−8.36 to−6.56)	0.78	<0.001
IL-6 (pg/mL)	12.94 ± 1.51	12.63 ± 1.44	12.72 ± 0.18	8.32 ± 0.18	−4.40 (−4.91 to −3.89)	0.80	<0.001

Data presented as mean ± standard deviation (SD) for baseline values and as adjusted mean ± standard error (SE) for 3-month outcomes. Between-group differences at 3 months were analyzed using ANCOVA, adjusted for baseline scores, age, and time since stroke. CI, Confidence Interval; ηp^2^, Partial Eta Squared; MMSE, Mini-Mental State Examination; MoCA, Montreal Cognitive Assessment; TMT, Trail Making Test; SS-QOL, Stroke-Specific Quality of Life; BDNF, Brain-Derived Neurotrophic Factor; TNF-α, Tumor Necrosis Factor-alpha; IL-6, Interleukin-6.

### Correlation analyses

**Figure 3:**
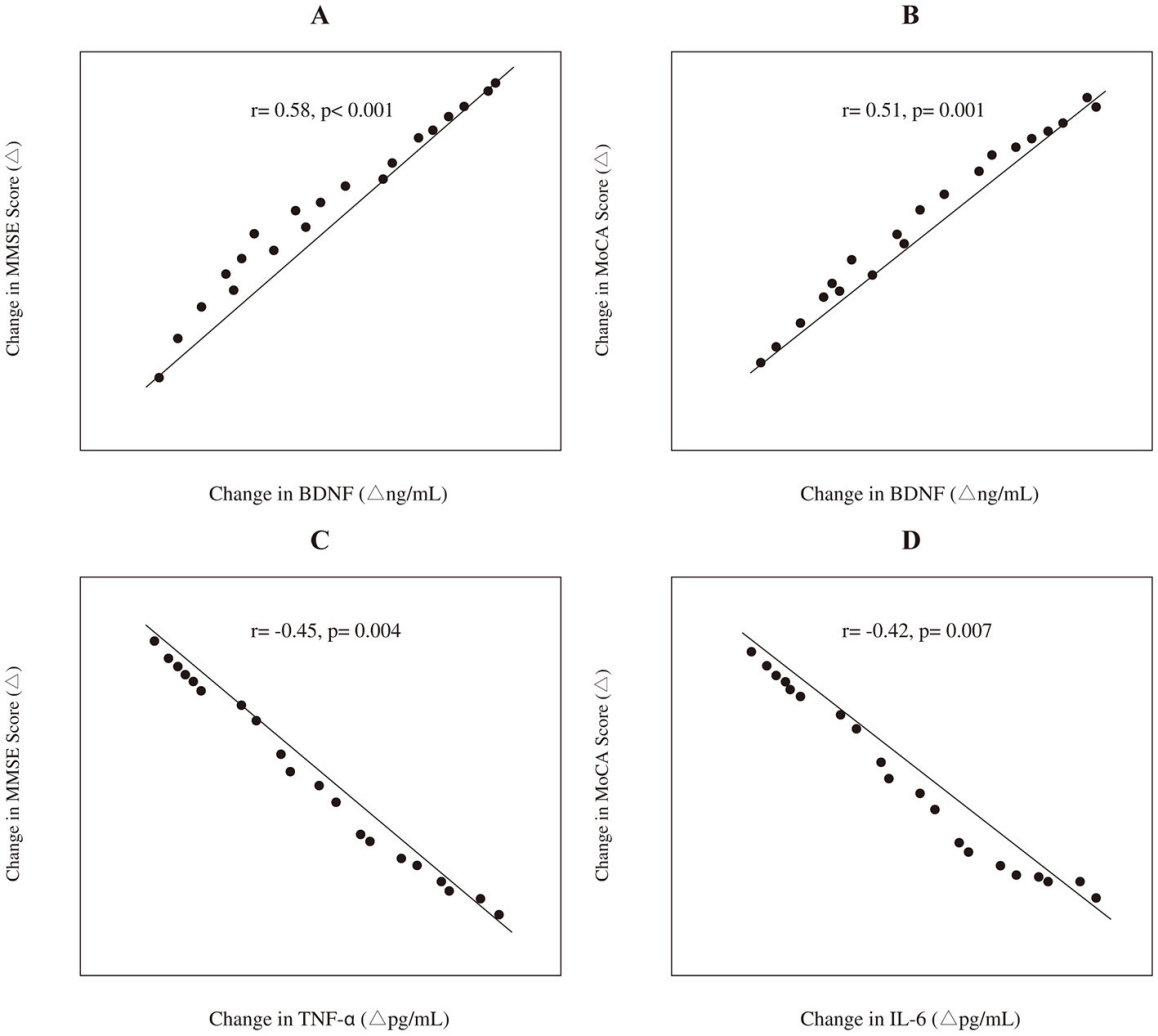
Correlations between the changes (Δ) in biochemical markers and cognitive scores in the experimental group (*n* = 40). The panels depict: **(A)** the positive correlation between Δ BDNF and Δ MMSE score; **(B)** the positive correlation between Δ BDNF and Δ MoCA score; **(C)** the negative correlation between Δ TNF-α and Δ MMSE score; and **(D)** the negative correlation between Δ IL-6 and Δ MoCA score. Trend lines and Pearson correlation coefficients (*r*) with *p*-values are shown.

In the experimental group, Pearson correlation analysis was conducted to explore the relationship between changes in biochemical markers and changes in cognitive scores from baseline to 3 months. The change (Δ) in BDNF levels showed a significant positive correlation with the change in MMSE scores (*r* = 0.58, *p* < 0.001) and MoCA scores (*r* = 0.51, *p* = 0.001). Conversely, the change in TNF-α levels was negatively correlated with the change in MMSE scores (*r* = −0.45, *p* = 0.004), and the change in IL-6 levels was negatively correlated with the change in MoCA scores (*r* = −0.42, *p* = 0.007). These correlations are graphically depicted in Figure 3.

## Discussion

This randomized, double-blind, sham-controlled trial demonstrated that a 4-week course of iTBS applied to the left DLPFC, combined with conventional therapy, significantly improved cognitive function, quality of life, and associated biochemical markers in patients with subacute post-stroke cognitive impairment. These improvements were sustained at a 3-month follow-up and were superior to those observed in the sham group.

Our findings align with and extend the results of previous studies. While recent systematic reviews have highlighted iTBS efficacy in post-stroke motor recovery (9, 10), evidence for cognitive benefits has been less conclusive, often stemming from trials limited by small samples (e.g., *n* = 20–30) and short follow-ups (≤6 weeks) (14, 15). For example, a 2023 systematic review (1) identified only six RCTs (total *n* = 312) evaluating iTBS for cognitive deficits, with heterogenous protocols and inconclusive outcomes. Our study contributes significantly to the field by employing a larger cohort (*n* = 80), a longer follow-up period of 3 months, robust double-blinding, and a multidimensional assessment battery including both cognitive and quality-of-life metrics alongside mechanistic biomarkers. This comprehensive approach provides stronger, more reliable evidence for iTBS as a viable tool for cognitive rehabilitation.

The mechanisms underlying these benefits likely involve dual pathways. First, the significant increase in serum BDNF in the iTBS group supports the hypothesis that iTBS enhances neuroplasticity. BDNF is a crucial mediator of synaptic plasticity, learning, and memory, and its upregulation is thought to facilitate neural repair and functional reorganization after brain injury (21–23). The positive correlation we observed between increased BDNF and improved cognitive scores further strengthens this link. Second, the reduction in pro-inflammatory cytokines TNF-α and IL-6 suggests an anti-inflammatory effect. Neuroinflammation is a key contributor to secondary brain injury after stroke, and its attenuation is associated with better neurological outcomes (24). Our finding that reduced inflammatory markers correlated with cognitive gains suggests that iTBS may help create a more favorable microenvironment for neural recovery.

The significant improvements in SS-QOL scores underscore the holistic benefits of iTBS, extending beyond objective cognitive metrics to patients' perceived daily functioning and emotional wellbeing. Furthermore, the magnitude of these improvements is clinically significant. For instance, the mean 4.91-point increase in MMSE scores in the iTBS group surpasses the 3- to 5-point threshold often considered a notable clinical difference in stroke populations (25). For clinical practice, iTBS presents a promising, non-invasive adjunctive therapy that can be integrated into multidisciplinary rehabilitation programs. Its safety, tolerability, and short session duration enhance its feasibility in routine clinical settings.

Strengths of our study include its randomized, double-blind, sham-controlled design, a well-defined patient population in the subacute stroke phase, a standardized intervention protocol, and the inclusion of multidimensional outcomes. However, some limitations must be acknowledged: (1) The study was conducted at a single center, which may limit the generalizability of the findings. (2) The 3-month follow-up period, while longer than many previous trials, does not allow for conclusions about the long-term sustainability of the observed effects. (3) Heterogeneity in stroke lesion locations, which were not systematically mapped, may influence iTBS responsiveness. Future neuroimaging studies are needed to explore how lesion site and size modulate treatment effects. (4) We did not conduct subgroup analyses based on stroke type (ischemic vs. hemorrhagic) due to sample size constraints within each subgroup, which limits our understanding of differential effects. (5) While our sham stimulation was designed to mimic the auditory and sensory experience of active iTBS, we did not formally assess the success of participant blinding (e.g., via a post-intervention questionnaire). Therefore, the potential for incomplete blinding cannot be entirely excluded and should be addressed in future trials. 6) Finally, while the changes in BDNF, TNF-α, and IL-6 are compelling, these peripheral biomarkers are non-specific and can be influenced by external factors such as diet, physical activity, and stress, which were not rigorously controlled outside of the standardized in-patient rehabilitation. Future studies could benefit from including additional markers (e.g., C-reactive protein) and controlling for these potential confounders more strictly.

In future, large-scale, multicenter trials with longer follow-up periods (≥12 months) are needed to confirm our findings and validate the long-term efficacy of iTBS. Combining iTBS with advanced neuroimaging techniques (e.g., fMRI, DTI) and electrophysiology could help elucidate dose-response relationships and identify optimal stimulation targets for individual patients.

## Conclusion

This study provides robust evidence that iTBS is a safe and effective adjunctive intervention for improving cognitive function and quality of life in patients with subacute post-stroke cognitive impairment. By appearing to modulate both neuroplasticity and neuroinflammation, iTBS offers a promising dual-action therapeutic strategy. Future research should focus on optimizing treatment protocols and exploring its mechanisms further to facilitate its translation into standard clinical practice.

## Data Availability

The original contributions presented in the study are included in the article/supplementary material, further inquiries can be directed to the corresponding authors.
